# Analysis of Altered Flowering Related Genes in a Multi-Silique Rapeseed (*Brassica napus* L.) Line zws-ms Based on Combination of Genome, Transcriptome and Proteome Data

**DOI:** 10.3390/plants12132429

**Published:** 2023-06-23

**Authors:** Liang Chai, Haojie Li, Xiaoguang Zhao, Cheng Cui, Benchuan Zheng, Ka Zhang, Jun Jiang, Jinfang Zhang, Liangcai Jiang

**Affiliations:** 1 Crop Research Institute, Sichuan Academy of Agricultural Sciences, Chengdu 610066, China; 2 Environment-Friendly Crop Germplasm Innovation and Genetic Improvement Key Laboratory of Sichuan Province, Chengdu 610066, China; 3 Hybrid Rapeseed Research Center of Shaanxi Province, Yangling 712100, China

**Keywords:** frame-shift InDel, multi-silique trait, MADS-box protein, non-synonymous SNP

## Abstract

Based on previous researches, we further investigated the multi-silique trait in rapeseed (*Brassica napus* L.) line zws-ms. In this study, we used a relatively comprehensive list of flowering related genes in rapeseed and compared them between zws-ms and its near-isogenic line (NIL) zws-217. Genes were studied on genome, transcriptome and proteome levels and then we focused on genes with non-synonymous single nucleotide polymorphism (SNP) or frame-shift insertion-deletion (InDel), finding some genes on the list which changes their sequences. Then, combined with their annotation and the information of their orthologs, certain genes such as BnaA09g05900D, ortholog of *AGAMOUS-LIKE 42* (*AGL42*), which encodes an MADS-box protein, were assumed as probably responsible for the multi-silique trait. Also, we analyzed the Differentially Accumulated Proteins (DAPs) between zws-ms and zws-217, revealing some genes involved in homologous recombination and mismatch repair pathways. Since the development of flowers/siliques is crucial to crops and it influences the yield of rapeseed, this study paved a way to deeply understand the mechanism of the multi-pistil flower formation, which may facilitate researches on rapeseed production in future.

## 1. Introduction

Rapeseed (*Brassica napus* L.) is a major source of edible oil and biofuel [1] globally. The correct flower structure and successive silique (fruit) development are vital for this crop. As reported, silique number is crucial to seed yield per plant [2]. We previously reported zws-ms [3,4,5,6], a rapeseed material which had aberrant flowers with three pistils. Those abnormal flowers then developed into multi-silique trait, i.e., three independent siliques on one carpopodium.

In some other plants, like wheat (*Triticum aestivum* L.) [7,8,9,10,11,12], rice (*Oryza sativa* L.) [13,14] and sweet cherry (*Prunus avium* L.) [15,16], similar phenomena was discovered and described as “multi-pistil”. Yang [10] reported a three-pistil mutant controlled by a dominant gene on chromosome arm 2DL; then they constructed an F_2_ population from CM28 (normal pistil) × CM28TP (three-pistil), and then fine-mapped the three-pistil gene (*Pis1*) by using genotyping-by-sequencing (GBS) and Kompetitive Allele-Specific PCR (KASP) markers successively, followed by listing a series candidate genes nearest to *Pis1* with the assistance of gene annotations. Meanwhile, Zheng et al. [13] studied a rice mutant, *multi-floret spikelet 3* (*mfs3*), which had two pistils in a spikelet. The *mfs3* was crossed with sterile line 56S and 426 individuals in F_2_ populations were then used to map the *MFS3* gene, which then located in a region of 106.3 kb length on Chromosome 6. Afterwards, the expression levels of some spikelet development-related genes were quantified. Thus, the *MFS3* gene was assumed to be the candidate gene.

These multi-pistil-similar traits from various crops were not only morphologically described, but some of the causal genes have been mapped to chromosomal regions or even cloned, such as *FLORAL ORGAN NUMBER1* (*FON1*) [14,17], *FON4* (also known as *FON2*) [18,19], *LATERAL FLORET1* (*LF1*) [20] in rice and *PaMADS3*, *PaMADS4* and *PaMADS5* in sweet cherry [15]. However, so far, few underlying genes have been precisely cloned and functionally validated in *Brassica* plants. There is only one exception: the *BnFul*, which is the ortholog of *Fruitful* (*FUL*), was unexpectedly found causing the multi-silique in some of the transgenic *Arabidopsis* lines [21]. Thus, there is insufficient exiting information concerning multi-pistil trait in *B. napus*. Nevertheless, Wang et al. [22] theoretically summarized all the flowering-related genes in rapeseed, including the ones involved in flower development and apical meristem response, and this would provide an excellent reference to identify the potential underlying genes for the multi-silique trait.

In our earlier studies [3,4,5,6], we identified two associated regions for the multi-silique trait by using Whole Genome Re-sequencing (WGR) based on the construction of near-isogenic lines (NILs) zws-ms (multi-silique) and zws-217 (normal silique). Combined with transcriptome sequencing and gene annotation, some candidate genes were screened out. However, due to the complexity of its allotetraploid genome (AACC, 2n = 38) and limited data on genome and transcriptome level, precise casual genes have not been certified.

Proteins are executors of gene functions and represent types of life activities. The concept of proteome was first proposed in early 1990s [23,24]. Proteome analysis identifies all proteins translated in specific cells or tissues and their expression models. It serves as an important supplement to studies based on genome or transcriptome level. To date, it has been widely applied in many aspects of crop researches. Proteome analysis can serve to research the unintended side effects of the transgenic host by transgene insertions: Liu et al. [25] compared the differentially-accumulated proteins among *Bt*-transgenic *B. napus* and its hybrids with *Brassica juncea* L., as well as parental *B. napus* and *B. juncea*, and the results showed that unintended effects due to transgene flow were negligible and in the scope of natural variability of hybridization. This technology can also be used for defense mechanism research. Cao et al. [26] inoculated *B. napus* leaves with *Sclerotinia sclerotiorum* (Lib.) de Bary wild-type strain, nonpathogenic mutant strain and an empty agar plug (as the control), and then conducted their proteome analysis. After comparison of data from different treatments, they utilized the Gene Ontology (GO), Kyoto Encyclopedia of Genes and Genomes (KEGG) and protein-protein interaction prediction analysis, finding new defense mechanisms against *S. sclerotiorum*, which revealed that redox homeostasis, lipid signaling, calcium signaling, etc., contribute to the defense. In addition, in other crops, e.g., wheat, proteomic analysis facilitates the investigation of effect on silver nanoparticles by inorganic and organic chemicals [27]. Moreover, proteome also helped to investigate the effects on yield and nutritional qualities of soybean (*Glycine max* L.) sprout treated by extra Ca^2+^ [28].

In the present study, we further investigated the molecular mechanism for the multi-silique morphology on proteome level. We compared protein expression of zws-ms with zws-217 sampled from Xindu, where the two lines distinguished from each other in multi-silique trait, and Ma’erkang where the two lines both showed normal siliques. The numbers and types of expressed proteins were studied. Based on the annotations and the comparison with the known flowering related genes, some DAPs were screened out and some of them were supposed to underlie this trait. Gene annotations were used to help identify candidate genes and understand its mechanism.

## 2. Results

### 2.1. Identification of Differentially Accumulated Proteins between zws-ms and zws-217 in Two Environments

**Figure 1:**
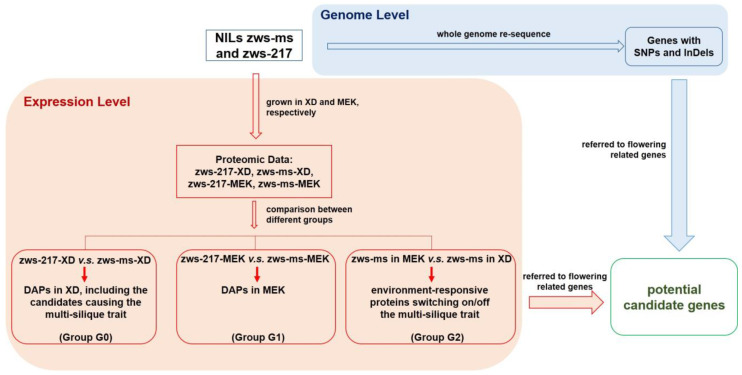
Schematic workflow of experimental design and comparisons of DAPs (Differentially Accumulated Proteins) from different samples.

Proteomic analysis was performed to identify proteins which were expressed differentially between zws-ms and zws-217, and between plants from the two environments, Xindu and Ma’erkang, on a holistic level. Protein was extracted from their budding stage, when the two lines initiated the morphological varieties in the number of the pistils and stamens in each flower. Proteins with ≥ 1.2-fold change for expression level and *p* ≤ 0.05 was defined as DAPs. The reliability of the proteomic data was validated by the box plot depicting coefficient of variation for proteins (Figure S1). The comparison of protein expression levels between zws-217 (as control) and zws-ms from Xindu was assigned as Group G0; the DAPs between the two lines grown in Ma’erkang as assigned as Group G1; while the DAPs in line zws-ms under the two environments was assigned as Group G2 (Figure 1). The overlapped proteins of different groups, as well as the specific proteins in each group, were then analyzed. Thus, 13,128 proteins were successfully quantified, and among them, 400 showed significant changes in their expression level between the two lines in the two environments.

**Figure 2:**
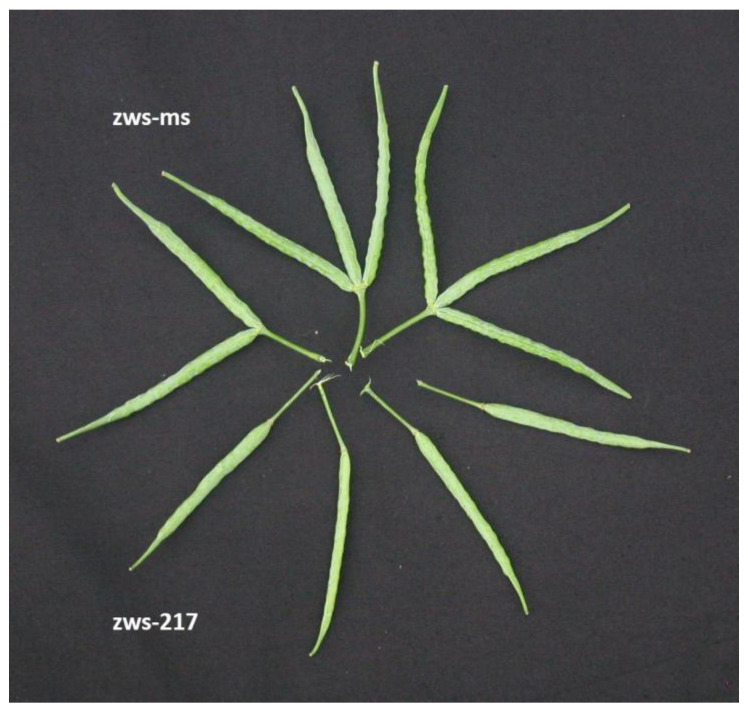
The phenomenon of multi-silique. In normal plant zws-217, there was only one silique on each carpopodium; where as in zws-ms, there were three siliques developed on each carpopodium.

As described earlier, zws-ms displayed multi-silique trait stably in Xindu (Figure 2), whereas both lines showed all normal siliques in Ma’erkang, where the climate is quite different.

The comparison between zws-217 (as control) and zws-ms (Table 1) from Xindu was assigned as G0, thus a total of 66 DAPs were identified in two lines from Xindu, where zws-ms and zws-217 distinguished from each other in the appearance of multi-silique trait. In particular, 41 proteins were up-regulated in zws-ms, while 25 proteins were down-regulated (Figure S2).

The comparison between zws-217 and zws-ms from Ma’erkang, where both lines represented normal floral organs and consequently regular siliques, was assigned as G1. Samples in G1 were also studied and 153 DAPs were found, consisting of 90 up-regulated proteins and 63 down-regulated ones (Figure S3). Moreover, the DAPs in zws-ms under the two environments were also analyzed, the comparison between Ma’erkang and Xindu was assigned as G2, and it was found that 214 proteins varied their abundances (Figure S4) between Xindu and Ma’erkang, among which 77 were up-regulated in zws-ms while 137 were down-regulated (Table 1).

### 2.2. Comparison of DAPs among Different Groups and Their Annotations

**Figure 3:**
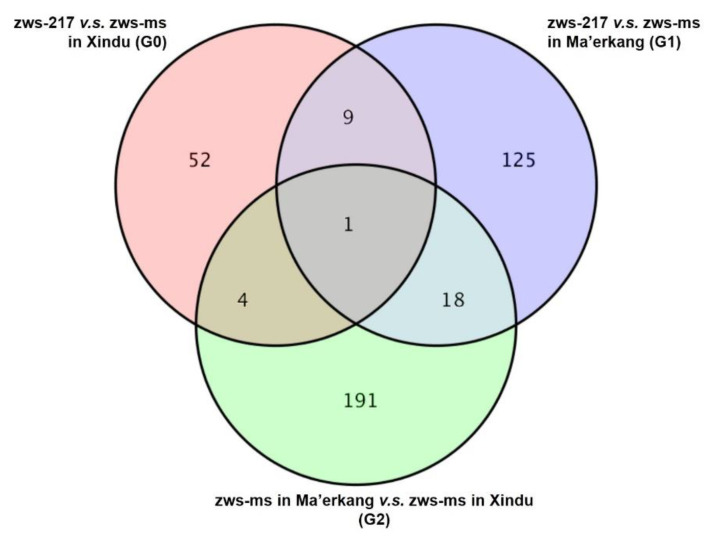
Venn diagram showing the numbers of specific DAPs and overlapped DAPs of G0, G1 and G2 groups.

Based on the analysis of DAPs in G0, G1 and G2, a Venn diagram was constructed to show the overlapped and specific DAPs of different comparisons (Figure 3). Only one DAP (BnaC03g32950D) was found simultaneously in three groups. Group G0 and G1 shared ten (9 + 1) DAPs, while G0 and G2 had five (1 + 4) common DAPs. Moreover, it was found that 52, 125 and 191 DAPs were specific in G0, G1 and G2 group, respectively.

**Figure 4:**
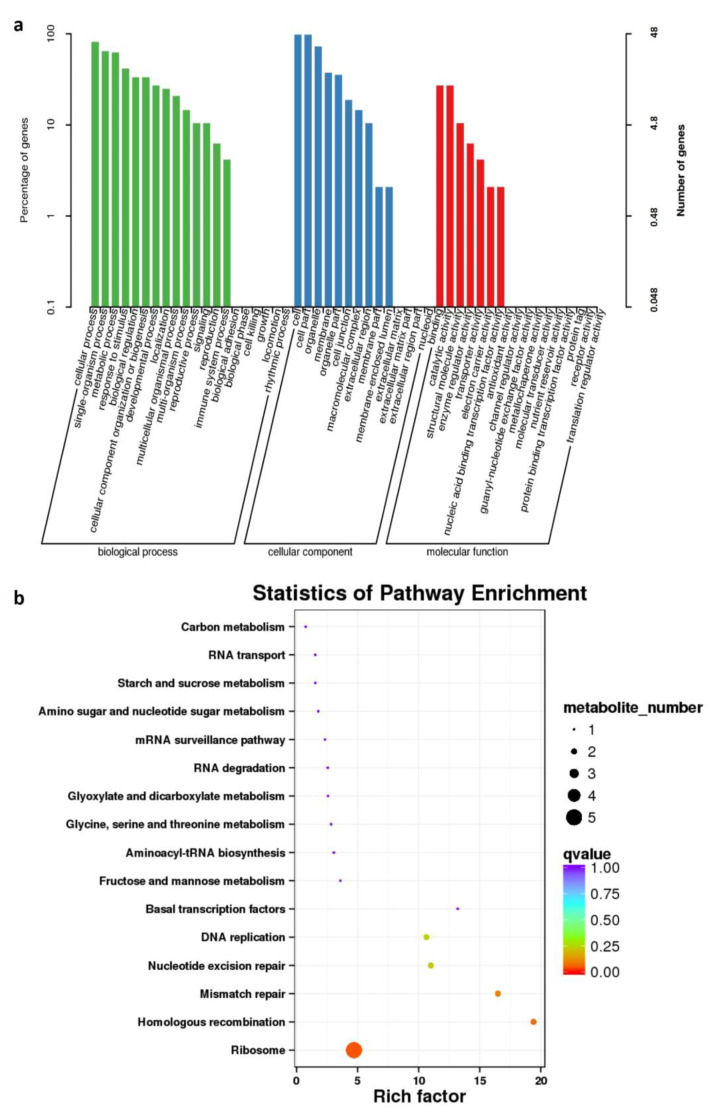
Annotation and enrichment of the 52 specific DAPs (Differentially Accumulated Proteins) in G0 group. (**a**) GO (Gene Ontology) classification of these 52 DAPs; (**b**) KEGG (Kyoto Encyclopedia of Genes and Genomes) enrichment of these 52 DAPs.

Among all the detected proteins, 12558 of them obtained GO annotations, while 4620 proteins were involved in KEGG annotations. Particularly, the specific 52 DAPs in G0, 17 of them were found in Chromosome A09 and C08, in which we identified two associated regions [5], accounting for 32.69% of DAPs in G0. As for the GO annotation, the term containing most DAPs were cell (GO:0005623) and cell part (GO:0044464). Cell (GO:0005623) term involved 47 DAPs, which consisted of 26 up-regulated proteins and 21 down-regulated ones (Figure 4, Table S1); while cell part (GO:0044464) term contained 26 and 21 proteins with their expression level enhanced or weakened in zws-ms, respectively. Moreover, the KEGG pathway showed that homologous recombination (ko03440) obtained the highest enrichment factor at 19.4 with *p*-value = 0.0045, involving two DAPs, BnaA09g48050D and BnaC08g49820D (Figure 4b; Table S1). Mismatch repair (ko03430) got the second highest enrichment factor at 16.5, which consisted of the same two DAPs.

Under the Ma’erkang conditions, zws-ms differed from zws-217 in 153 proteins, containing 125 specific proteins in this group. Thus, in G1 group (Table S2), there were 72 up-regulated specific DAPs and 53 down-regulated ones. While in the group of G2, 191 specific DAPs were discovered (Table S3). However, there is no enrichment of annotation for genes in either of the two groups, which implied that there was rarely functional commonalities among these DAPs in the two groups.

### 2.3. Identification of the DAPs and DEGs Related to Flowering

When compared with the 1173 flowering related genes (Table S4) summarized by Wang et al. [22], there is no common genes found within the 52 DAPs in G0. In other words, no known flowering related gene on the list varied its protein expression level between zws-ms and zws-217 in Xindu. However, in G2 group, where zws-ms showed DAPs between two environments, one flowering related gene, BnaAnng35580D, was identified in the multi-silique line. Moreover, when we compared the 129 DEGs varying between zws-ms and zws-217 in Xindu, which were described in our previous research [5], with all the listed flowering related genes, we screened out one of them; that is, BnaAnng35580D, again. It was found this gene reduced its transcriptional level in zws-ms.

Further study showed that BnaAnng35580D was involved in “RNA processing and modification”. Its ortholog, AT2G21660 in Arabidopsis, is *GLYCINE RICH PROTEIN 7* (*GRP7*). AT2G21660 is involved in autonomous pathway [22], and it encodes a small glycine-rich RNA binding protein that is part of a negative-feedback loop through which it regulates the circadian oscillations of its own transcript (https://www.arabidopsis.org/servlets/TairObject?id=32851&type=locus, accessed on 1 May 2023).

### 2.4. Discovery of the Flowering-Realted Genes with Non-Synonymous SNP or Frame-Shift InDel in Line zws-ms

Totally, out of those 1173 flowering related genes (Table S4) which were summarized by Wang et al. [22], there were four genes identified with non-synonymous SNP(s) (Table 2): BnaA09g05900D, BnaC02g42040D, BnaC08g36330D and BnaA09g57260D.

BnaA09g05900D from the multi-silique line zws-ms displayed a non-synonymous mutation site at 2880166 bp on Chromosome A09. It represented 84.6% amino acid identity with its ortholog in *Arabidopsis*, *AT5G62165*, which encodes a MADS-box protein, AGAMOUS-like 42 (AGL42). BnaC08g36330D had two non-synonymous sites in zws-ms varying from zws-217 or reference genome; according to its annotation, it encodes a transcription activator and it is homologous to *GOLDEN2-LIKE 1* (*GLK1*, also known as *GPRI1*) in *Arabidopsis*.

BnaC02g42040D and BnaA09g57260D had one non-synonymous SNP, respectively, in line zws-217 rather than zws-ms.

As for the InDels, three genes, *BnaA02g28220D*, *BnaA09g57260D* and *BnaAnng33220D* (Table 3), were found obtaining frame-shift insertions or deletions. Specifically, *BnaA02g28220D*, which is ortholog of AT3G26744, was identified as *INDUCER OF CBF EXPRESSION 1* (*ICE1*) and had three insertions in line zws-ms, compared with zws-217 and reference Darmor. BnaA09g57260D in zws-ms obtained an insertion at 4114345 bp and a deletion at 4114386 bp, its ortholog AT1G10120 encodes a basic helix-loop-helix (bHLH) DNA-binding superfamily protein. BnaAnng33220D’s ortholog, AT2G19520, was related to late flowering.

Out of these genes, BnaA09g05900D represented 84.6% amino acid identity with *AGL42* (AT5G62165), and its annotation involves flower development and apical meristem. Based on this, BnaA09g05900D was considered the most possible candidate gene for the multi-silique trait in zws-ms herein.

## 3. Discussion

In this study, the flowering related genes in the multi-silique rapeseed line zws-ms were analyzed on genomic, transcriptomic and proteomic levels. Previously, we constructed NILs, compared zws-ms and ms-217 and carried out the WGR, finding out SNPs and InDels between the two lines, and DEGs were also analyzed [5]. However, due to lack of referential genes, we just selected some potential underlying genes based on the annotations, as well as their orthologs’ information. That is because there were few genes known controlling the multi-silique (or multi-pistil) trait to date, especially in *Brassica* plants, though the phenomenon has been discovered and described in several species, such as wheat [7,8,9,10,11,12], rice [13,14] and sweet cherry [15,16] as mentioned above. Afterwards, Wang et al. [22] published a comprehensive list of flowering-relevant genes in rapeseed, which facilitated our current investigation greatly; thus, based on that data, we studied non-synonymous SNPs or frame-shift InDels anew, by referring them to those genes with relatively clear functions regarding to flowering, particularly the development of flower or number of floral organ; in addition, proteome was also analyzed.

When we compared the proteomic data between zws-ms and zws-217, it showed that DAPs on A09 and C08 accounted for 32.69% of all the DAPs, of which the rate was considerably higher than any other chromosomes. This indicated that the majority of DAPs were concentrated on the associated regions we identified before [5]. In the consequent investigation, proteins from the two lines in the two environments were divided into various groups, and comparisons between them were made and analyzed, which was then represented by the Venn diagram (Figure 3). Each part in that diagram displayed a unique contrast, standing for some specific DAPs in certain conditions. To illustrate, certain DAPs out of the 52 specific proteins in G0 group, where the NILs distinguished from each other in the appearance or absence of the multi-silique trait, are more likely to be the causal genes for this phenomenon. Meanwhile, G2 group indicated the comparison of proteins in zws-ms plants grown in two environments, respectively; in other words, these proteins changed their expression level in Ma’erkang, where the multi-silique trait disappeared; thus, some of them were supposed to be regulated by environmental factors and consequently switched off the trait. In addition, it is assumed that DAPs in G1 group, which varied between the two lines in Ma’erkang, were related to the fragments on the chromosomes that harboring the underlying genes. That is to say, they might be just physically closed to the causal genes. That is why the DAPs in G1 group could not be enriched in any pathways.

Meanwhile, it is advisable to look up genes in G0 and G2 classes, especially those flowering related ones. When analyzing the KEGG pathway for the DAPs in G0 group, we noticed that the pathways with the highest enrichment factors were homologous recombination (ko03440) and mismatch repair (ko03430). This trend is understandable in view of the fact that distant hybridization not only gathers traits from parental plants, but also creates certain new traits. For instance, in early generations of resynthesized *Brassica napus*, exchange of homologous chromatid arms and even chromosome rearrangement occurred, leading to novel phenotypic traits [29,30,31,32]. Specifically, as mentioned above, the zws-ms also originated from *Brassica napus* × *Brassica rapa*. Hence, it is assumed that the crossing led to some mutations involving homologous recombination and mismatch repair processes, and certain genes among them were perceived to serve these mismatch-repairing process. Therefore, it led to the genes highly enrich in these relevant pathways. Nevertheless, which genes/proteins are the precise ones underlying the multi-silique phenomenon still needs further investigation. In addition, we noticed that BnaAnng35580D was up-regulated in zws-ms when planted in Xindu, compared to its expression level in Ma’erkang. This implied a potential negative correlation with the multi-silique trait. Thus, we paid attention to its ortholog in *Arabidopsis*, *GLYCINE RICH PROTEIN 7* (*ATGRP7* or *CCR2*) to further analyze its functions. *AtGRP7* is controlled by the circadian clock and was shown to regulate flowering time dominantly by affecting *FLOWERING LOCUS C* (*FLC*), which is an MADS-box repressor [33], whereas the *atgrp7-1* mutant showed obvious late flowering [33]; thus, it is seen that *AtGRP7* regulates flowering time in *Arabidopsis*. As for MADS-box proteins, they will be discussed latter on.

In our earlier report, two associated chromosomal regions were identified, harboring more 2044 genes within them [5]. Although we analyzed the SNPs and InDels, as well as the annotations of these genes, and primarily predicted some candidate genes, it is not easy to further shorten the list of the real causal genes, because there was little useful information which can efficiently guide. In fact, a deep understanding of the flowering related genes in rapeseed can play a crucial role in the orientation for the consequent direction. Fortunately, a relatively complete list of 1173 genes by Wang et al. [22] facilitated our research considerably, which concludes all genes involved in not only flowering time, but also the floral developments. Thus, these genes were subjected to the earlier data on genomic (SNPs and InDels) and transcriptome levels (DEGs), combined with the current discovery on proteome level.

As a result, it was showed that the multi-silique line zws-ms varied sequences in two genes, BnaA09g05900D and BnaC08g36330D, which resulted in theoretical non-synonymous mutations. Notably, BnaA09g05900D is ortholog of *AGL42* that encodes a MADS-box protein. The MADS-box genes are comprehensively found in various plant species, and they are responsible for control the development of inflorescence meristem, floral meristem, and floral organ identity [34]. *OsMADS1* [17], a MADS gene in rice, was found responsible for the recessive multi-pistil trait in mutant *mp3*. Besides, *PavFUL*, a MADS-box gene in sweet cherry that is homologous to *FRUITFUL* (*FUL*, also known as *AGL8*) in *Arabidopsis*, its seasonal expression level was found highly related to multi-pistil formation; furthermore, when overexpressed in *Arabidopsis*, it also caused in multi-silique phenomenon [34]. Interestingly, the multi-pistil trait in sweet cherry is highly influenced by temperature. Particularly, in warmer regions, the multi-pistil rate was higher in sweet cherry. Likewise, the trend in rapeseed zws-ms [5] greatly similar to this. Thus, they might share certain mechanism, involving some similar genes, specifically the MADS-box genes. In addition, it is initially known that *FUL* negatively regulates *SHATTERPROOF1* (*SHP1*) and *SHATTERPROOF2* (*SHP2*), and prevents formation of the dehiscence zone, thus generating the indehiscent fruits in the overexpressing transgenic *Arabidopsis* [35]. As the researches went further, it was revealed that *BnFUL* from rapeseed could not only enhance the pod shattering resistance in transgenic *Arabidopsis*, but it also led to multi-silique trait in some of the lines unexpectedly [36]. Thus, it is seen that *AGL* genes were relevant to the number of pistil in several plants, thought the mechanism is still elusive. Based on the aforementioned information, we assume that BnaA09g05900D, an MADS-box protein ortholog to AGL42, in which one non-synonymous took place in zws-ms in this study, was a potential candidate gene that altered its sequence and may consequently contribute to the multi-silique trait in zws-ms.

BnaC08g36330D is the ortholog of AT2G20570, which encodes GOLDEN2-LIKE 1 (GLK1) protein, involved in autonomous pathway, regulating flowering time. Recent study showed that it contributes to pod photosynthesis and affects seed yield-related traits in *Arabidopsis* [37]; however, there has been no clue to indicate the correlation to flower morphology.

When it comes to the flowering related genes with frame-shift InDel, three genes were identified herein. BnaA02g28220D, ortholog of AT3G26744 (*INDUCER OF CBP EXPRESSION 1*, *ICE1*), encodes a MYC-like bHLH transcriptional activator that binds specifically to the MYC recognition sequences in the CBF3 promoter. It is involved in vernalization pathway, regulating the flowering time. AT1G10120 is homologous to BnaA09g57260D, and it encodes a basic helix-loop-helix (bHLH) DNA-binding superfamily protein named *CRY2-INTERACTING BHLH 4* (*CIB4*); to our best knowledge, it mediated flowering time via CRY2 [38]. As for BnaAnng33220D, its ortholog AT2G19520 is identified as *MULTICOPY SUPPRESSOR OF IRA1 4* (*MSI4* or *FVE*), and it is involved in flowering time [39]. So, it can be seen that the three genes take part in controlling the flowering time rather than the flower morphology.

Apart from the genomic level, the flowering related genes were also investigated on proteomic level. In the Venn diagram (Figure 3), where DAPs from different comparisons were categorized, the specific 52 DEGs in G0 group were also worth of studying, since they directly represented the causal factors that are highly related to the multi-silique trait. Although we did not find any flowering related genes in this group, in other words, there was no flowering related genes showing significant difference in protein expression level between zws-ms and zws-217, we noticed that these DAPs were most enriched in homologous recombination (ko03440) and mismatch repair (ko03430) for KEGG pathways. This information implies that the crossing might be related. Intriguingly, the multi-silique phenomenon in zws-ms did originate from descendants of *B. napus* × *B. rapa*, as we reported [5]. Thereby, the concerned genes, like BnaA09g48050D and BnaC08g49820D, are perceived to carry out some repairing mismatch repair due to the *B. napus* × *B. rapa* cross. In fact, many multi-pistil traits in various plants attributes to crossing. For instance, *mp3*, a rice mutant displaying an inconstant number of pistils ranging from one to four pistils in a floret, was obtained from a cross of indica- and japonica- rices [17]. Thus, it is assumed that crossing between species or subspecies is a way to generate the abnormal floral structure. It is undeniable that the multi-pistil formation in plants is more complex than we expected. Besides the genetic mutation [40] or crossing [17], heterogeneous cytoplasm [8,41] or environmental factors like temperature [15] also influence this trait.

To date, we have known that multi-pistil underlying genes belong to several types: (1) *FLORAL ORGAN NUMBER 1* (*FON1*), ortholog of *CLAVATA1* (*CLV1*) in *Arabidopsis*, encodes a leucine-rich repeat receptor-like kinase (LRR-RLK) [14,17]. (2) *FON2*, also known as *FON4* [18,19], belongs to the CLE family. (3) *LATERAL FLORET 1* (*LF1*) encoded a class III homeodomain-leucine zipper (HD-ZIP III) protein [20]. (4) Whereas in dicotyledon sweet cherry, *PaMADS3, PaMADS4* and *PaMADS5* encode proteins belonging to the MADS family [15]. In this study, we analyzed the flowering related genes in multi-silique rapeseed zws-ms on genome, transcriptome and proteome levels, and found an MADS-box gene, BnaA09g05900D, changes its sequence compared with zws-217 or reference genome. Based on our knowledge of AGL genes, we assume BnaA09g05900D may be involved in the multi-silique formation in zws-ms.

It must be added, however, that this study is, to some extent, on the basis of the list by Wang et al. [22], and although it is relatively comprehensive, it may still miss some genes. That is because the list is based on the known flowering genes in *Arabidopsis* and the alignment to rapeseed genome, but there are still many genes which may relate to floral structure in fact but their functions are not explored yet. Thus, more studies are still on going.

## 4. Materials and Methods

### 4.1. Plant Materials

The seeds of rapeseed near-isogenic lines (NILs), zws-ms (multi-silique) and zws-217 (normal silique), originated from cultivar Fuguo, were kept in the Crop Research Institute, Sichuan Academy of Agricultural Sciences. In Xindu, where zws-ms displayed multi-silique, the two lines were sew simultaneously in September 2021; while in Ma’erkang, where both lines show only normal siliques, they were grew in early June 2022.

### 4.2. The Identification of the Flowering Related Genes in Rapeseed

The collection of the flowering-related genes by Wang et al. [21] was used as the database, in which the researchers totally identified in *B. napus* 1173 flowering-related genes classified into nine types: aging pathway, ambient temperature, autonomous pathway, clock and photoperiod pathway, flower development and apical meristem response pathway, flowering time integrator, hormones pathway, sugar signal and vernalization. The Differentially Accumulated Proteins (DAPs), the Differentially Expressed Genes (DEGs) and the genes with sequence mutation mentioned below were compared with these 1173 genes, and then analyzed with annotations further.

### 4.3. Protein Extraction and Digestion

Buds from three individuals from each line were collected at budding stage as our earlier description [5]. Samples from each plant were ground respectively, and then 300 μL of 8M urea and protease inhibitor were successively added into each sample. After centrifuged at 14,100× *g* for 20 min, the supernatant was collected. The protein concentration was determined according to the method proposed by Bradford [42] and then was stored at −80 °C.

The proteins samples were then subjected to reduction with 100 mM dithiothreitol (DTT) for 1 h at 30 °C, and subsequently were alkylated with sufficient iodoacetamide (IAM) for 1 h at Room Temperature (RT) in the dark. Alkylated proteins were digested with trypsin (trypsin: protein = 1: 50) overnight at 37 °C. Finally, the sample were all desalted with C18 cartridge to remove the urea, followed by vacuum centrifugation.

### 4.4. Liquid Chromatography Tandem Mass Spectrometry Analysis

The Liquid Chromatography Tandem Mass Spectrometry (LC-MS/MS) analysis was carried out as described by Shen et al. [43]. There was 41 μL of tandem mass tag (TMT) reagent (ThermoFisher Scientific Inc., Waltham, MA, USA) added to 100 μg of the digested product. Then the mixture was oscillated, centrifuged and incubated at RT for 1 h, and then incubated in 5% quenching reagent for 15 min to terminate the reaction. The samples were stored at −80 °C after lyophilization.

Each fraction was injected for nano LC-MS/MS analysis. The peptide mixture was loaded onto the C18-reversed phase analytical column in buffer A (0.1% Formic acid) and separated with a linear gradient of buffer B (80% acetonitrile and 0.1% Formic acid) at a flow rate of 300 nl/min.

LC-MS/MS analysis was performed on a Q Exactive Plus mass spectrometer (Thermo Fisher Scientific) that was coupled to Easy nLC (Thermo Fisher Scientific) for 90 min. The mass spectrometer was operated in positive ion mode. MS data was acquired using a data-dependent top10 method dynamically choosing the most abundant precursor ions from the survey scan (350–1800 *m*/*z*) for HCD fragmentation. Survey scans were acquired at a resolution of 70,000 at *m*/*z* 200 with an AGC target of 3e6 and a maxIT of 50 ms. MS2 scans were acquired at a resolution of 35,000 for HCD spectra at *m*/*z* 200 with an AGC target of 5 × 10^4^ and a maxIT of 45 ms, and isolation width was 2 *m*/*z*. Dynamic exclusion for selected ions was 30 s. Normalized collision energy was 30 eV.

### 4.5. Differentially Accumulated Proteins (DAPs) and Differentially Expressed Genes (DEGs) Identification

Software Proteome Discoverer2.4 was used as described by Li et al. [44] in order to process the data generated. Trypsin/P was specified as the cleavage enzyme. The parameters were set as follows: mass tolerance for precursor ion and mass tolerance for product ion were 15 ppm and 0.02 Da, respectively. Carbamidomethyl residues on Cys were specified as the fixed modification; while the oxidation on Met, the acetylation of the N-terminus and TMT 10-plex of tyrosine and lysine were specified as variable modifications. The false discovery rate (FDR) was adjusted to <1%. Proteins with ≥1.2-fold change for expression level and *p* ≤ 0.05 calculated by Student’s *t*-test were identified as Differentially Accumulated Proteins (DAPs). As mentioned above, the comparisons of protein expression levels between were showed in Figure 1. The overlapped proteins of different groups (Figure 3), as well as the specific proteins in each group, were then analyzed.

As for the Differentially Expressed Genes (DEGs) [45], the data of our previous report [5] was used.

### 4.6. The Functional Annotations and Analysis of the Proteins

Gene Ontology (GO) annotations for proteins identified were derived from the UniProt database, which classify GO annotations into three general categories: biological process (BP), cellular component (CC) and molecular function (MF) [46]. Enrichment analysis of these annotations was conducted by a *t*-test. The Kyoto Encyclopedia of Genes and Genomes (KEGG) database (https://www.genome.jp/kegg/, accessed on 1 March 2023) was used to perform the enrichment analysis of pathways [47]. Above analyses was performed using BMKCloud (www.biocloud.net, accessed on 1 March 2023).

### 4.7. The Analysis of SNPs and InDels in the Flowering Related Genes on Genomic Level

As previously described [5], single nucleotide polymorphisms (SNPs) and insertion-deletions (InDels) were identified by combining the results of analysis with GATK [48] and SAMtools [49] via SNP or InDel calling, with the default parameters, after aligned to the *B. napu*s reference genome V4.1 (http://www.genoscope.cns.fr/brassicanapus/data/, accessed on 1 March 2023). Furthermore, SNPs and InDels varied between the two lines, zws-ms and zws-217, were regarded as polymorphic and were then used to analyze with emphasis. Genes varying between zws-ms and zws-217 or reference species Darmor, especially those that cause non-synonymous SNPs or frame-shift InDels in zws-ms, were emphasized. Their annotations, as well as their orthologs in model plant *Arabidopsis*, were further analyzed.

## 5. Conclusions

In this study, we found certain genes varying their sequences or expression levels between NILs zws-ms and zws-217. Out of these altered genes, we noticed a MADS-box gene, BnaA09g05900D, which was identified as *AGL42* ortholog, was specifically noteworthy, since many of its family members, like *AGL8* (*FUL*), *PaMADS3*, *PaMADS4* and *PaMADS5*, were found responsible for the formation of multi-pistil in several species. In addition, the KEGG information showed that DAPs were most enriched in pathways concerning homologous recombination (ko03440) and mismatch repair (ko03430), which is in line with the fact that zws-ms was originated from *B. napus* × *B. rapa*, which may lead to some exchange of homologous chromatid arms and even chromosome rearrangement. Hence, this research has compensated for some deficiencies of our previous studies, and we take a further step forward finding the precise underlying genes for the multi-silique trait in rapeseed line zws-ms.

## Figures and Tables

**Table 1:** The number of differentially accumulated proteins between zws-ms and zws-217 in two environments.

Comparison	Total DAPs	Up-Regulated DAPs in zws-ms	Down-Regulated DAPs in zws-ms
zws-217 v.s. zws-ms in Xindu (G0)	66	41	25
zws-217 v.s. zws-ms in Ma’erkang (G1)	153	90	63
zws-ms in Ma’erkang v.s. zws-ms in Xindu (G2)	214	77	137

**Table 2:** The four genes with non-synonymous SNPs between zws-ms and zws-217.

Gene ID	Position	Ref	zws-ms	zws-217	Swissprot Annotation	Ortholog in Arabidopsis	
						Gene ID	Description
BnaA09g05900D	2,880,166	C	Y (C/T)	C	MADS-box protein AGL42	AT5G62165	AGAMOUS-like 42 (AGL42)
BnaC02g42040D	44,852,629	G	G	R (A/G)	Protein LEO1 homolog	AT5G61150	VERNALIZATION INDEPENDENCE 4 (VIP4)
BnaC08g36330D	33,648,610	A	R (A/G)	A	Transcription activator GLK1	AT2G20570	GBF’s pro-rich region-interacting factor 1 (GPRI1)
	33,648,961	A	G	R (A/G)			
BnaA09g57260D	4,114,551	A	A	R (A/G)	Transcription factor bHLH74	AT1G10120	basic helix-loop-helix (bHLH) DNA-binding superfamily protein

**Table 3:** The three genes with frame-shift mutations between zws-ms and zws-217.

Gene ID	Pos	Ref	zws-ms	zws-217	Swissprot Annotation	Ortholog in Arabidopsis	
						Gene ID	Gene ID
BnaA02g28220D	20,796,604	G	G,GAGAGGGAGAGCA	G	Transcription factor ICE1	AT3G26744	INDUCER OF CBF EXPRESSION 1 (ICE1)
	20,796,652	A	A,AACC	A			
	20,796,686	C	C,CTGTAACATG	C			
	20,797,054	CTT	CTT	CTT,C			
BnaA09g57260D	4,114,345	G	GTAA	G,GTAA	Transcription factor bHLH74	AT1G10120	basic helix-loop-helix (bHLH) DNA-binding superfamily protein
	4,114,386	GT	G	GT,G			
	4,115,144	AT	AT	AT,A			
	4,115,529	C	C	C,CCCA			
BnaAnng33220D	37,892,579	C	CGTCTCCTCAAGGG	C,CGTCTCCTCAAGGG	WD-40 repeat-containing protein MSI4	AT2G19520	FVE

## Data Availability

The datasets generated and analyzed during the current study are available from the corresponding author upon reasonable request.

## Supplementary Materials

The following supporting information can be downloaded at: https://www.mdpi.com/article/10.3390/plants12132429/s1, Figure S1: The box plot depicting coefficient of variation for proteins; Figure S2: The heat map of DAPs between zws-ms and zws-217 in Xindu; Figure S3: The heat map of DAPs between zws-ms and zws-217 in Ma’erkang; Figure S4: The heat map of DAPs between zws-ms in Xindu and zws-ms in Ma’erkang; Table S1: The 52 specific DAPs in G0 group and their annotations; Table S2: The 125 specific DAPs in G1 group and their annotations; Table S3: The 191 specific DAPs in G2 group and their annotations; Table S4: The list of the 1173 flowering related genes in rapeseed.
