# Supplementary figures and images for: Analysis of Altered Flowering Related Genes in a Multi-Silique Rapeseed (Brassica napus L.) Line zws-ms Based on Combination of Genome, Transcriptome and Proteome Data
Source: Plants (Basel). 2023 Jun 23;12(13):2429. doi: 10.3390/plants12132429 (PMC10346454; doi:10.3390/plants12132429)

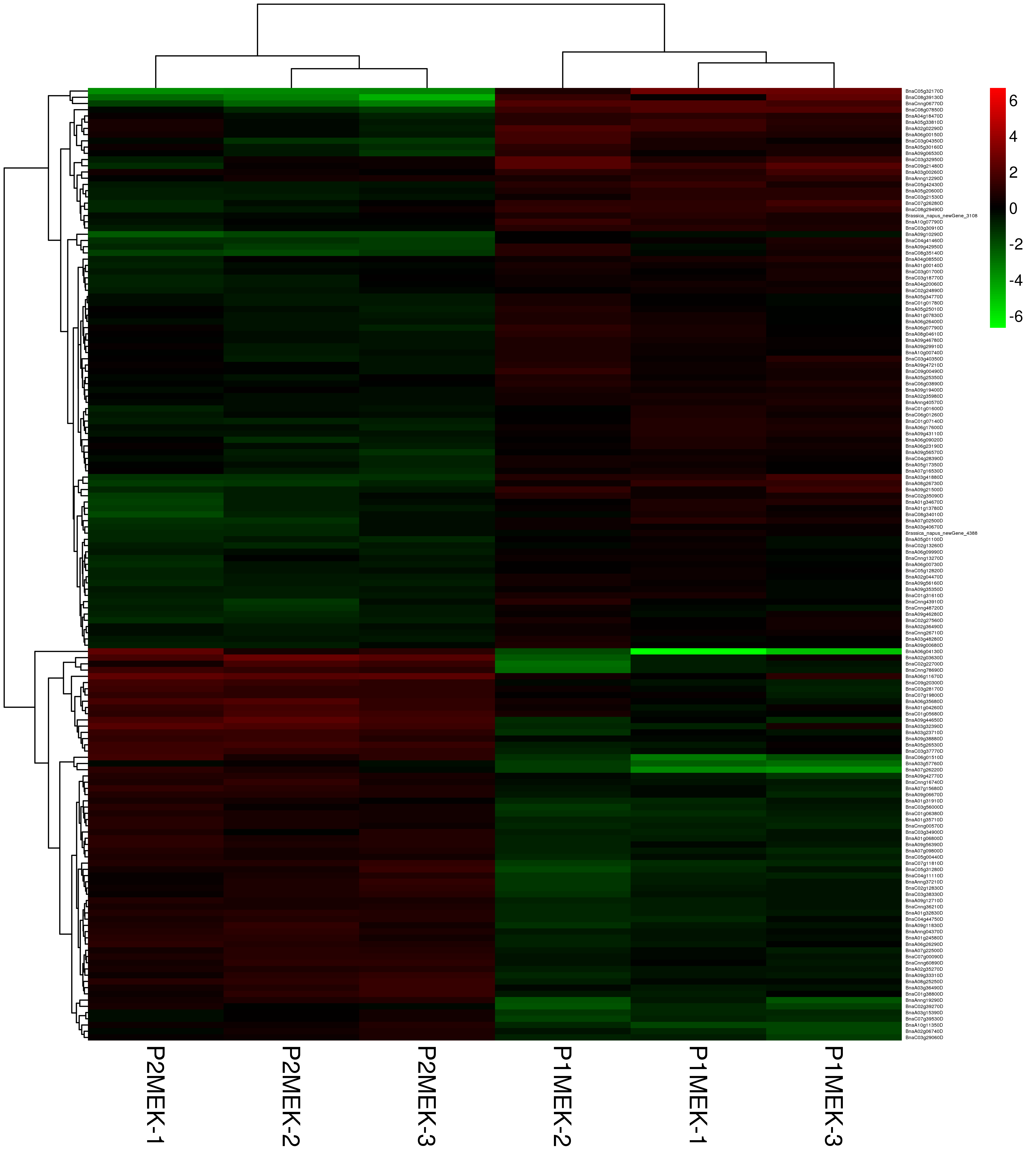

Supplement: Supplementary file 1 [file plants-12-02429-s001.zip › figure S3-heat map-MEK.png]

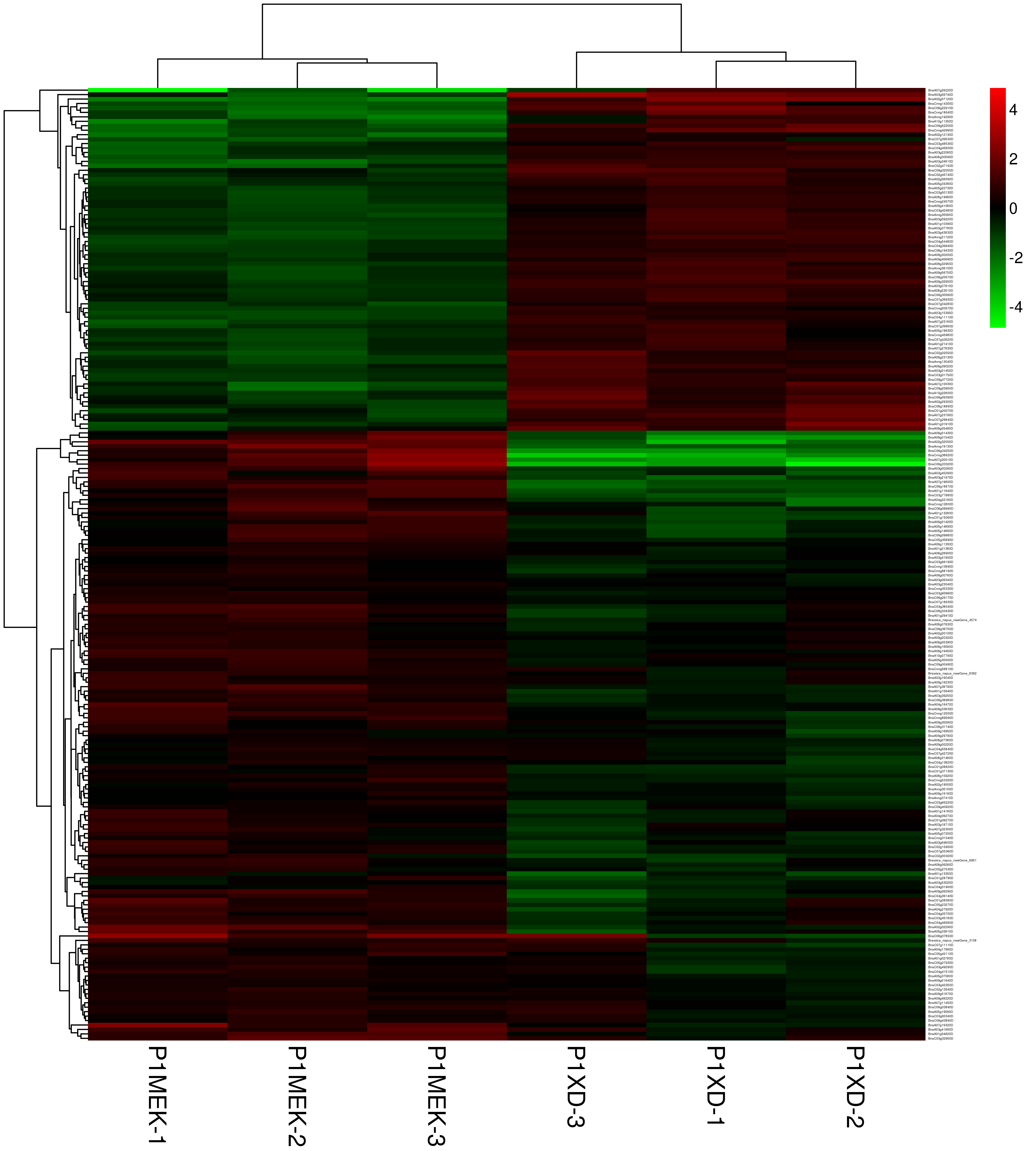

Supplement: Supplementary file 1 [file plants-12-02429-s001.zip › figure S4-heat map xd-p1-mek-p1.png]

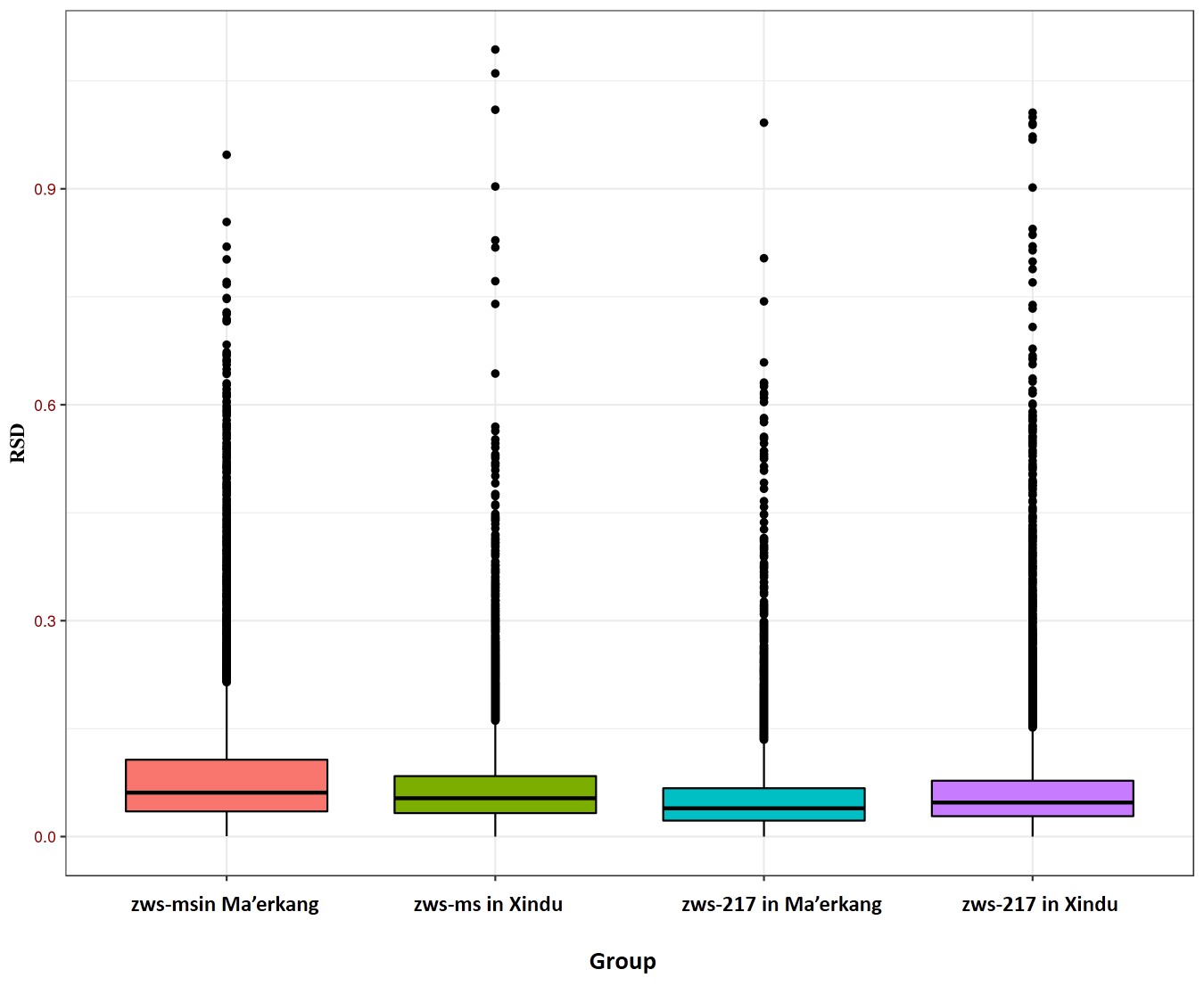

Supplement: Supplementary file 1 [file plants-12-02429-s001.zip › figure S1-new.jpg]

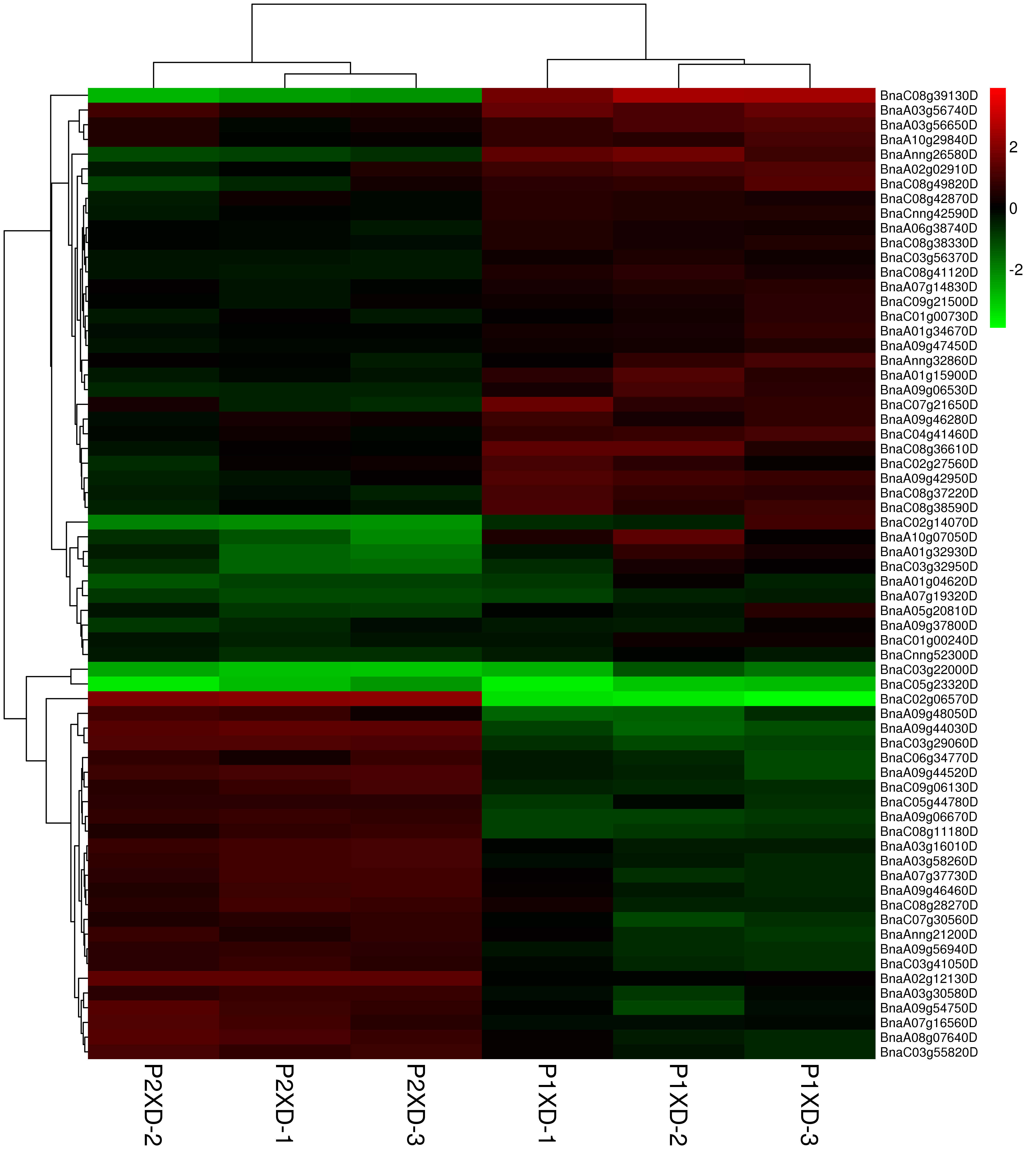

Supplement: Supplementary file 1 [file plants-12-02429-s001.zip › figure S2-heat map-XD.png]
